# IL28B Gene Polymorphism SNP rs8099917 Genotype GG Is Associated with HTLV-1-Associated Myelopathy/Tropical Spastic Paraparesis (HAM/TSP) in HTLV-1 Carriers

**DOI:** 10.1371/journal.pntd.0003199

**Published:** 2014-09-18

**Authors:** Tatiane Assone, Fernando Vieira de Souza, Karen Oliveira Gaester, Luiz Augusto Marcondes Fonseca, Olinda do Carmo Luiz, Fernanda Malta, João Renato Rebello Pinho, Fernanda de Toledo Gonçalves, Alberto Jose da Silva Duarte, Augusto Cesar Penalva de Oliveira, Jorge Casseb

**Affiliations:** 1 Laboratório de Dermatologia e Imunodeficiências, Departamento de Dermatologia, Faculdade de Medicina da Universidade de São Paulo, São Paulo, São Paulo, Brazil; 2 Instituto de Medicina Tropical de São Paulo, São Paulo, São Paulo, Brazil; 3 Instituto de Doenças Infecciosas “Emilio Ribas” (IIER) de São Paulo, São Paulo, São Paulo, Brazil; 4 Departamento de Medicina Preventiva, Faculdade de Medicina da Universidade de São Paulo, São Paulo, São Paulo, Brazil; 5 Laboratório de Imuno-hematologia e Hematologia Forense – LIM40, Departamento de Medicina Legal, Ética Médica, Medicina Social e do Trabalho, Faculdade de Medicina da Universidade de São Paulo, Sao Paulo, São Paulo, Brazil; Hospital Universitário, Brazil

## Abstract

**Background:**

The polymorphisms of IL28B have been described as important in the pathogenesis of infections caused by some viruses. The aim of this research was to evaluate whether IL28B gene polymorphisms (SNP rs8099917 and SNP rs12979860) are associated with HAM/TSP.

**Methods:**

The study included 229 subjects, classified according to their neurological status in two groups: Group I (136 asymptomatic HTLV-1 carriers) and Group II (93 HAM/TSP patients). The proviral loads were quantified, and the rs8099917 and rs12979860 SNPs in the region of IL28B-gene were analyzed by StepOnePlus Real-time PCR System.

**Results:**

A multivariate model analysis, including gender, age, and HTLV-1 DNA proviral load, showed that IL28B polymorphisms were independently associated with HAM/TSP outcome in rs12979860 genotype CT (OR = 2.03; IC95% = 0.96–4.27) and in rs8099917 genotype GG (OR = 7.61; IC95% = 1.82–31.72).

**Conclusion:**

Subjects with SNP rs8099917 genotype GG and rs12979618 genotype CT may present a distinct immune response against HTLV-1 infection. So, it seems reasonable to suggest that a search for IL28B polymorphisms should be performed for all HTLV-1-infected subjects in order to monitor their risk for disease development; however, since this is the first description of such finding in the literature, we should first replicate this study with more HTLV-1-infected persons to strengthen the evidence already provided by our results.

## Author Summary

New evidence has shown that the pathogenic mechanism of disease-associated HTLV-1 infection is an impairment of the immunity. More recently, it has been demonstrated that IL28B polymorphisms are more likely to occur among HTLV-1 infected subjects and are associated with higher proviral loads in HTLV-1 carriers. Based on anti-HCV properties exhibited by IL28B, we examined the possibility of an association between IL28B polymorphisms (rs8099917 and rs12979860 SNPs) and HAM/TSP occurrence in a large cohort of HTLV-1-infected subjects in Sao Paulo city, Brazil. This study included 229 HTLV-1-infected subjects classified according to their neurological status in two groups (asymptomatic vs HAM/TSP cases), and observed that persons with SNP rs8099917 genotype GG and rs12979860 genotype CT may present a distinct immune response against HTLV-1 infection. Thus, it seems reasonable to suggest that a search for IL28B polymorphisms should be performed for all HTLV-1-infected subjects in order to monitor their risk for HAM/TSP development.

## Introduction

Human T-cell lymphotropic Virus Type 1 (HTLV-1) is a retrovirus etiologically linked to adult T-cell leukemia/lymphoma [1], HTLV-1-associated myelopathy/tropical spastic paraparesis (HAM/TSP) and other inflammatory diseases [2]. This virus is endemic in Japan, Caribbean Basin, and some countries in Latin America [3], [4]; fifteen to 25 million people are estimated to be infected worldwide [5], [6]. In Brazil, the highest prevalence of HTLV-1 is found in the Northeastern region, particularly in the cities of Sao Luiz and Salvador [7].

New evidence has shown that the pathogenic mechanism of disease-associated HTLV-1 infection is an impairment of the immunity [8]. More recently, it has been demonstrated that IL28B (also known as interferon lambda 3) polymorphisms are more likely to occur among HTLV-1infected subjects and that the IL28B polymorphisms are associated with higher proviral loads in HTLV-1 carriers [9].

IL28B is located on chromosome 19, using a cytokine receptor that is specific to the IL10Rβ and IL28Rα, which is expressed in macrophages, dendritic cells and hepatocytes. The signaling receptors for interferons are the result of phosphorylation of JAK1 and Tyk2 kinases, which actively transcribe the factor containing STAT1, STAT2 and interferon regulatory factor 9. Those groups of interferons up regulate viral infections by other interferons, leading to an immune response to HCV [10], [11]. Based on anti-HCV properties exhibited by IL28B we designed the current study aim to examine the possibility of an association between IL28B polymorphisms (rs8099917 and rs12979860 SNPs) and HAM/TSP in a large cohort of HTLV-1-infected subjects in Sao Paulo city, Brazil.

## Materials and Methods

The HTLV outpatient clinic from Institute of Infectious Diseases “Emilio Ribas” (IIER) has been following HTLV-infected patients for 15 years, 229 of whom were studied in this project. For the purpose of this study, only patients who are more than 18 years old and were in active follow-up during the period from June 2011 to May 2012 were invited to participate.

The Ethical Board of the IIER approved the protocol (Number 13/2011), and an informed signed consent was obtained from all participants prior to study inclusion. The subjects were classified according to their neurological status in two groups: Group I (136 asymptomatic HTLV-1 carriers) and Group II (93 HAM/TSP patients). Blood samples were collected in acid-citrate-dextrose solution, and PBMC separated by Ficoll density gradient centrifugation (Pharmacia, Uppsala, Sweden). Cells were washed with saline solution; cell number was adjusted to 2×10^6^ cells and then stored at −80°C. DNA was extracted using a commercial kit (Illustra Tissue and Cells Genomic Prep Mini Spin kit, Easton Turnpike, Fairfield, CA) according to manufacturer's instructions. After this procedure the DNA was stored at −80°C for later analysis.

### Quantification of HTLV-1 proviral load

The HTLV-1 proviral load was quantified by real-time PCR, using primers and probes targeting the *pol* gene: SK110 (5'-CCCTACAATCCAACCAGCTCAG-3', HTLV-1 nucleotide 4758-4779 (GenBank accession No. J02029), and SK111 (5'-GTGGTGAAGCTGCCATCGGGTTTT-3', HTLV-1 nucleotide 4943-4920). The internal HTLV-1 TaqMan probe (5'-CTTTACTGACAAACCCGACCTACCCATGGA-3') was selected using the Oligo (version 4, National Biosciences, Plymouth, MI, USA) and Primer Express (Perkin-Elmer Applied Biosystems, Boston, MA, USA) software programs and checked through a search of GenBank. The probe was located between positions 4829 and 4858 of the HTLV-1 genome and carried a 5' reporter dye FAM (6-carboxy fluorescein) and a 3' quencher dye TAMRA (6-carboxy tetramethylrhodamine). For quantification of the human albumin gene, the primers Alb-S (5'-GCTGTCATCTCTTGTGGGCTGT-3') and Alb-AS (5'-AAACTCATGGGAGCTGCTGGTT-3')and the albumin TaqMan probe (5'-FAMCCTGTCATGCCCACACAAATCTCTCCTAMRA-3') were used as described previously [12], [13]. Albumin DNA was quantified jointly in all samples in order to determine the amount of DNA used as an endogenous reference to normalize variations due to differences in PBMC counts or DNA extraction. The 25-µl PCR mixture for HTLV-1 or albumin DNA amplifications consisted of 5 µl DNA extract, primers SK110 and SK11 or Alb-S and Alb-AS (10nM of each), 10 nM HTLV-1 or albumin TaqMan probe, TaqMan Universal Master Mix II (Applied Biosystems, Foster City, CA). For both the HTLV-1 and albumin DNA amplifications, after one cycle at 50°C for 2 min and one cycle at 95°C for 10 min, a two-step PAC procedure was used consisting in 15 s at 95°C and 1 min at 60°C for 45 cycles. Amplifications were carried out using the ABI 7300Fast Real-Time PCR System (Applied Biosystems, Foster City, CA). The HTLV-1 copy number in each clinical sample was estimated by interpolation from the plasmid regression curve. To determine the proviral load, the HTLV-1 DNA copy number was normalized to the amount of the cellular albumin of the clinical sample, which was quantified in parallel. All samples were run in duplicates. Results were expressed as HTLV-1 DNA copies/10^4^ PBMCs, as described elsewhere [13]. Based on the median of asymptomatic individuals, 200 copies/10^4^ PBMCs of PVL was the value used as a cut off to discriminate from HAM/TSP subjects.

### Testing for rs8099917 and rs12979860 SNP Genotyping

The rs8099917 and rs12979860 SNPs in the region of IL28B-gene were analyzed by StepOnePlus Real-time PCR System (ABI TaqMan allelic discrimination, Applied Biosystems, Foster City, CA) with the help of a Custom TaqMan SNP Genotyping Assay developed together with Applied Biosystems [14]. Also, TaqMan Universal PCR Master Mix (Applied Biosystems, Foster City, CA) was used. All samples were run in duplicated, and negative and positive controls for all variations of each SNP regions were also included in the analysis.

### Statistical analysis

Statistical analysis was conducted using Student's t-test for parametric data, and the chi-square test for proportions. Possible differences in patient characteristics or laboratory values among the groups were evaluated with two-way Mann-Whitney's test and Kruskal-Wallis test. Group analysis was done using Anova test (GraphPadSoftware 5.0, La Jolla, CA). Bivariate logistic analysis was performed to identify independent variables associated with HAM/TSP. Variables associated with the outcome at a significance level of p<0.20(HAM/TSP) in the bivariate analysis were included in a multivariate logistic model; the only exception to this procedure was the inclusion of the variable gender, which was included regardless of its statistical significance in the bivariate model. Logistic analysis was performed with the aid of Stata 10 software (StataCorp. 2009. *Stata: Release10*. Statistical Software. College Station, TX). The x^2^ G test for “Goodness of Fit” was used to verify whether the proportions of the polymorphisms were unequally distributed in Hardy-Weinberg equilibrium (HWE)[15].

## Results

One hundred and thirty six asymptomatic HTLV-1-infected subjects and 93 HAM/TSP patients were studied. The mean age of the study population was 52 years, and 154 (67.25%) were female (Table 1). Sexual risks of transmission were not different for HAM/TSP and asymptomatic patients. IL28B genotype distribution at the SNP rs12979860 in HTLV-1 patients was as follows: CC (n = 68; 30.49%), CT (n = 139, 62.33%) and TT (n = 16; 7.17%), six patients had no DNA enough to carried out the tests (Table 1). Median HTLV-1 DNA proviral load (PVL) from asymptomatic HTLV-1 subjects was 36 copies, whereas the PVL median from HAM/TSP patients was 236 copies/10^4^ PBMC. Furthermore, the IL28B CT genotype was more frequent in HAM/TSP patients than in asymptomatic carriers (p = 0.1067). IL28B genotype distribution at the SNP rs8099917 was: TT (n = 144, 64.0%), GT (n = 63, 28.0%) and GG (n = 18; 8.0%), four patients had no DNA enough to carried out the tests (Table1). TT genotype was more frequent in HAM/TSP patients when compared to asymptomatic carriers (p = 0.2879). Bivariate analysis revealed that the proviral load was significantly associated with HAM/TSP (p<0.01) as well as with the polymorphisms SNP rs8099917 in the profile GG (p<0.01) and SNP rs12979860 in the profile CT (p = 0.01) (Table 1).

**Table 1:** Bivariate analysis of IL28B polymorphisms and HTLV-1-associated myelopathy.

Characteristic	Total N(%)	Asymptomatic N(%)	HAM/TSP N(%)	OR (95%CI)	P Value
**Male**	75(32.75)	45 (33.0)	30 (38.8)	-	-
**Female**	154 (67.25)	91 (67.00)	63 (61.2)	1.083 (0.5916–1.823)	0.99
**Age>50 years**	142 (62.01)	76 (55.9)	66 (71.0)	1.93 (1.101–3.383)	0.03
**Age≤50 years**	87 (37.99)	60 (44.1)	27 (29.0)	-	-
**HTLV-1 DNA proviral Load**					
**<200**	142 (62.01)	99 (72.8)	43 (46.2)	-	-
**≥200**	87 (37.99)	37 (27.2)	50 (53.8)	3.11 (1.785–5.423)	<0.01
**SNP rs12979860** *					
**CC**	68 (30.49)	48 (36.9)	20 (21.5)	1	
**CT**	139 (62.33)	69 (53.1)	70 (75.3)	2.45 (1.22–4.93)	0.01
**TT**	16 (7.17)	13 (10.0)	03 (3.2)	0.41 (0.08–2.09)	0.28
**SNP rs8099917** **					
**TT**	144 (64.00)	90 (68.2)	54 (58.1)	1	
**TG**	63 (28.00)	40 (30.3)	23 (24.7)	0.64 (0.33–1.23)	0.18
**GG**	18 (8.00)	02 (1.5)	16 (17.2)	7.00 (1.92–25.4)	<0.01

SNP: Single nucleotide polymorphism.

*Four patients without enough samples for IL28b rs8099917 assay.

**Six patients without enough samples for IL28b rs12979860 assay.

A multivariate model analysis, including gender, age, and HTLV-1 DNA proviral load, showed that IL28B polymorphisms were independently associated with HAM/TSP outcome in rs12979860 genotype CT (OR = 2.03; IC95% = 0.96–4.27) and in rs8099917 genotype GG (OR = 7.61; IC95% = 1.82–31.72). In this model, age was also significantly associated with the outcome when included as a continuous variable (Table 2). Results of the x^2^ test for “Goodness of Fit” were x^2^ 22.52, p<0.0001 for SNP rs12979860, and x^2^ 7.63, p = 0.005 for SNP rs8099917, revealing that deviation existed in the Hardy Weinberg equilibrium.

**Table 2:** Multivariate analysis for IL28B polymorphisms and HTLV-1-associated myelopathy outcome.

Characteristic	*OR (95% CI)*	*P value*
**Age (continuous)**	0.99 (0.99–1.00)	0.06
**Gender (male)**	0.90 (0.46–1.76)	0.76
**HTLV-1 DNA proviral Load**		
**<200**	1	
**≥200**	1.18 (0.62–2.25)	0.59
**SNP rs12979860**		
**CC**	1	
**CT**	2.03 (0.96–4.27)	0.06
**TT**	0.22 (0.03–1.40)	0.11
**SNP rs8099917**		
**TT**	1	
**GT**	0.69 (0.34–1.38)	0.30
**GG**	7.61 (1.82–31.72)	<0.01

SNP: Single nucleotide polymorphism.

## Discussion

The neuropathology of HAM/TSP provides evidence that immune mediated mechanisms may play a significant role in the pathogenesis of the disease [14], [16]. A few studies have looked for a possible association between Il28B polymorphisms – rs8099917 and rs12979860 and HAM/TSP outcome, while examining the HTLV-1 proviral load as a confounder; those who did have reported controversial results [17] [9], [18]. The major finding of this study, so far, was the independent association of IL28B polymorphism SNP rs8099917 (GG) with the development of HAM/TSP when compared to asymptomatic HTLV-1 carriers. To our knowledge, this is the first time that these findings have been related with HAM/TSP outcome, although polymorphism SNP rs12979860 has already been implicated in a sustained virological response to HCV infection in patients treated with pegylated interferon alpha and ribavirin [19], a finding that has not been demonstrated for other viral infections, such as HBV and HIV infections [20]. Moreover, the profile of polymorphism SNP rs12979860 could induce the production of IFN-lambda 3, and an immune active HTLV-1 infection with neuronal damage in the spinal cord, providing additional evidence for immune damage in the HAM/TSP pathogenesis [11], [16]. Our own previous study showed that patients with HAM/TSP had higher levels of IFN-gamma and MIP1-alpha production compared to asymptomatic patients [21]. However, and despite all the evidence mentioned above, we cannot exclude the possibility that those polymorphisms are but a marker of the disease.

The majority of the studies, so far, emphasized the polymorphism SNP rs12979860. However, Soriano's group showed higher proviral loads among HAM/TSP patients, although no association between HAM/TSP and IL28B has been found [10]. This fact could have been due to the small sample size (n = 12 patients) or to a different ethnic background of patients in that study. More recently, a Brazilian study also failed to find a positive association between the IL28B rs12979860 polymorphisms and an increased risk of developing HAM/TSP in 24 patients [18]. Our study population was not in HWE equilibrium for both polymorphisms, what may even indicate a higher risk for patients carrying the GG genotype. Furthermore, there seems to be biological plausibility for the association we found, based on previous findings [10], [18].

Thus, for the first time, this polymorphism was associated with clinical outcome, as described in our study. In fact, PVL is important in HAM/TSP patients since they may have up to 5–6 times more HTLV-1 proviral DNA in PBMC when compared to asymptomatic carriers, what may be related to many of the immunologic responses discussed above [22]. Other genetic background characteristics, such as some HLA haplotypes, have been described as involved in HAM/TSP pathogenesis [23] or pro-inflammatory factors [17]. In this regard, IL28B polymorphisms are influenced by genetic ancestry. Given the historical context of the Brazilian colonization, the population displays unique genetic characteristics, presenting phenotypes from African, European and Amerindian populations [24]. For example, the genotype GG (SNP rs8099917) genotype is more frequent in Asiatic, African and European Americans, while CC (SNP rs12979860) genotype is more frequent in European and Amerindian populations [25]. In recent study in South of Brazil, showed that in HCV-infected subjects and non-HCV-infected individuals, the higher allele frequency of rs12979860 C and rs8099917 T [26]. The distribution of those genotypes is similar to the genotype distribution found in our study.

Similarly, among HCV chronic patients, those who carried the G risk allele at rs8099917 had lower PBMC mRNA expression of IL28B [27]. It is likely that regulation will differ in the infected tissue and even between cell types within the liver and maybe in the spinal cord, as reported recently for some interferon stimulated genes [28]. IL28B attenuates IL-13; it is also possible that the cytokine produced in persons with the protective genotype diminishes IL-13 to a lesser extent than the molecule with the risk genotype, similar to the protective effect of the least inhibitory interactions between KIR and HLA-C [29]. Thus, IL28B and other type 3 interferons like IL28A or IL-29 trigger an antiviral cascade via JAK-STAT that is similar and probably synergistic with type 1 interferons (such as interferon alfa), although using distinct receptors, contributing to HAM/TSP immune pathogenesis.

Finally, a tentative explanation: persons with SNP rs8099917 genotype GG may present a distinct immune response against HTLV-1 infection. However, since this is the first description of this finding in the literature, we should first replicate this study with more HTLV-1-infected persons to strengthen the evidence already provided by our results. It is likely that IL28B may have some importance for protection against disease progression [11], but further cohort studies should be done in order to test this hypothesis.

## Supporting Information

Checklist S1 STROBE Checklist.
(DOCX)
