# Supplementary material for: IL28B Gene Polymorphism SNP rs8099917 Genotype GG Is Associated with HTLV-1-Associated Myelopathy/Tropical Spastic Paraparesis (HAM/TSP) in HTLV-1 Carriers
Source: PLoS Negl Trop Dis. 2014 Sep 18;8(9):e3199. doi: 10.1371/journal.pntd.0003199 (PMC4169378; doi:10.1371/journal.pntd.0003199)
Supplement: Checklist S1 — STROBE Checklist. (DOCX) [file pntd.0003199.s001.docx]

STROBE Statement—Checklist of items that should be included in reports of ***cross-sectional studies***

|  | Item No | Recommendation |
| --- | --- | --- |
| **Title and abstract** | 1 | (a)Prevalence of IL28B gene polymorphisms rs12979860 and rs8099917 genotypes among HTLV-1-infected individuals, regardless of clinical status. |
|  |  | (*b*) A total of 136 asymptomatic HTLV-1-infected subjects and 93 HAM/TSP patients were studied. The mean age of the study population was 52 years, and 154 (67.25%) were female. The IL28Bgenotype distribution at the SNP rs12979860 in HTLV-1 patients was as follows: CC (n=68; 30.49%), CT (n=139, 62.33%) and TT(n=16; 7.17%), six patients had no DNA enough to carried out the tests.Median HTLV-1 DNA proviral load (PVL) from asymptomatic HTLV-1 subjects was 36 copies, whereas the PVL median from HAM/TSP patients was236copies/10^4^ PBMC. Furthermore, the IL28B CT genotype was more frequent in HAM/TSP patients than in asymptomatic carriers (p=0.1067). The IL28Bgenotype distribution at the SNP rs8099917 was: TT (n=144, 64.0%), GT (n=63, 28.0%) and GG (n=18; 8.0%),four patients had no DNA enough to carried out the tests |
| Introduction | | |
| Background/rationale | 2 | It is estimated thatrange from 5 to10 millionHTLV-1 infected individuals worldwide and Brazil is considered a highly endemic area for HTLV-1 infection with the largest absolute number of HTLV-1 infected individuals, with more than one million people living with this virus. New evidence has shown that the pathogenic mechanism of disease-associated HTLV-1 infection is an impairment of the immunity. More recently, it has been demonstrated that IL28B (also known as interferon lambda 3) polymorphisms are more likely to occur among HTLV-1infected subjects and that the IL28B polymorphisms are associated with higher proviral loads in HTLV-1 carriers. |
| Objectives | 3 | The aim of this study is to examine the possibility of an association between IL28B polymorphisms (rs8099917 and rs12979860 SNPs)and HAM/TSPin a large cohort of HTLV-1-infected subjects in Sao Paulo city, Brazil.. |
| Methods | | |
| Study design | 4 | Cross-sectional study |
| Setting | 5 | From a total 450 HTLV-1-infected individuals, including asymptomatic carriers and HAM/TSP, 229 of them were consecutively evaluated forIL28B polymorphisms (rs8099917 and rs12979860 SNPs) from Jane 2011 to May 2012 from Sao Paulo city, Brazil |
| Participants | 6 | (*a*) HTLV-1 without co-infections |
| Variables | 7 | HIV or HCV infections were excluded, only older than 18 years old |
| Data sources/measurement | 8* | For each variable of interest, give sources of data and details of methods of assessment (measurement). Describe comparability of assessment methods if there is more than one group |
| Bias | 9 | The same researcher evaluated all subjects |
| Study size | 10 | 229 of them were consecutively evaluated forIL28B polymorphisms (rs8099917 and rs12979860 SNPs) from Jane 2011 to May 2012 from Sao Paulo city, Brazil |
| Quantitative variables | 11 | Explain how quantitative variables were handled in the analyses. If applicable, describe which groupings were chosen and why |
| Statistical methods | 12 | (*a*) Statistical analysis was conducted using Student’s t-test for parametric data, and the chi-square test for proportions. The x^2^ G test for “Goodness of Fit” was used to verify whether the proportions of the polymorphisms were unequally distributed in Hardy-Weinberg equilibrium (HWE). |
|  |  | (*b*) Possible differences in patient characteristics or laboratory values among the groups were evaluated with two-way Mann-Whitney’s test and Kruskal-Wallis test. Group analysis was done using Anova test (GraphPad Software 5.0, La Jolla, CA). |
|  |  | (*c*) Samples that haven’t DNA enough to run the tests, was excluded in statistical analysis. |
|  |  | (*d*) If applicable, describe analytical methods taking account of sampling strategy |
|  |  | (*e*) IC 95% ; p≤0.05 |
| Results | | |
| Participants | 13* | (a) The same researcher evaluated all subjects during the study |
|  |  |  |
|  |  |  |
| Descriptivedata | 14* | (a) HTLV-1+, adults >18 years; |
|  |  | (b) SNP rs12979860 in HTLV-1 subjects, six patients had no DNA enough to carried out the tests, and in the SNP rs8099917 four patients had no DNA enough to carried out the tests. |
| Outcome data | 15* | Report numbers of outcome events or summary measures |
| Main results | 16 | (*a*) Variables associated with the outcome at a significance level of p<0.20(HAM/TSP) in the bivariate analysis were included in a multivariate logistic model; the only exception to this procedure was the inclusion of the variable gender, which was included regardless of its statistical significance in the bivariate model. |
|  |  | (*b*) Report category boundaries when continuous variables were categorized |
|  |  | (*c*) If relevant, consider translating estimates of relative risk into absolute risk for a meaningful time period |
| Other analyses | 17 |  |
| Discussion | | |
| Key results | 18 | Subjects with SNP rs8099917 genotype GG (OR=7.00; IC95%= 1.92-25.40) and rs12979618 genotype CT (OR= 2.03; IC95%= 0.96-4.27) may present a distinct immune response against HTLV-1 infection. |
| Limitations | 19 | Relative low number of HAM/TSP patients |
| Interpretation | 20 | These findings indicate as IL28B polymorphisms in genotype TT (rs8099917) is a potential marker of HAM/TSP. |
| Generalisability | 21 | This is the first description of this finding in the literature, we should first replicate this study with more HTLV-1-infected persons to strengthen the evidence already provided by our results. |
| Other information | | |
| Funding | 22 | CNPq:134001/2011-7; FAPESP:2010/07076-4; FAPESP: 2012/23397-0 |

*Give information separately for exposed and unexposed groups.

**Note:** An Explanation and Elaboration article discusses each checklist item and gives methodological background and published examples of transparent reporting. The STROBE checklist is best used in conjunction with this article (freely available on the Web sites of PLoS Medicine at http://www.plosmedicine.org/, Annals of Internal Medicine at http://www.annals.org/, and Epidemiology at http://www.epidem.com/). Information on the STROBE Initiative is available at www.strobe-statement.org.
